# Topical RNA Interference Induces Mortality in the Cotton–Melon Aphid *Aphis gossypii* with No Adverse Effect on the Predator *Propylea japonica*

**DOI:** 10.3390/insects16030276

**Published:** 2025-03-05

**Authors:** Chong Zhan, Boya Jiao, Letian Xu, Yu Peng, Yao Zhao

**Affiliations:** 1 State Key Laboratory of Biocatalysis and Enzyme Engineering, School of Life Sciences, Hubei University, Wuhan 430062, China; zhanchong@stu.hubu.edu.cn (C.Z.); xuletian@hubu.edu.cn (L.X.); 2 Hubei Key Laboratory of Regional Development and Environmental Response, Faculty of Resources and Environmental Science, Hubei University, Wuhan 430062, China; 3 Faculty of Science, University of Sydney, Camperdown, NSW 2050, Australia; linerosa@163.com

**Keywords:** RNA interference, Topical RNAi, cotton–melon aphid, survival, ladybird beetle

## Abstract

The cotton–melon aphid (*Aphis gossypii* Glover), a globally distributed polyphagous pest, primarily infests cucurbit crops and leads to significant reductions in both crop yield and quality. Overreliance on chemical insecticides has resulted in widespread resistance development, highlighting the urgent requirement for alternative control strategies. This study evaluates the potential of topical RNA interference (RNAi) for managing cotton–melon aphids. We first analyzed instar-specific expression profiles of four candidate RNAi target genes (*ATPE*, *IAP*, *Cat*, and *ilvE*), employed topical dsRNA delivery to silence these genes, and subsequently evaluated their effects on aphid mortality, growth rates, and reproductive capacity. Furthermore, we investigated the non-target effects of RNAi-treated aphids on the predator ladybird beetles *Propylea japonica*. The results indicate that topical dsRNA delivery successfully silenced the target genes, significantly impairing aphid development and fecundity while inducing mortality, with no adverse effects on the beneficial predator. This method provides a powerful tool for insect gene functional studies and a promising solution for RNAi-based pest management.

## Simple Summary

The cotton–melon aphid (*Aphis gossypii* Glover) harms many types of plants, including cotton, melon, and other vegetables. Farmers have been using chemicals to control these pests, but the aphids are becoming resistant to these chemicals, making them harder to manage; so, there is a great need to find effective alternatives. RNA interference (RNAi) based on double-stranded RNA (dsRNA) delivery is a post-transcriptional gene silencing mechanism that has the potential to be a new insect control strategy. However, these applications are hampered by aphids due to the lack of effective dsRNA delivery methods. In this study, we explored an application of an RNAi method called topical dsRNA delivery to the cotton–melon aphid. We focused on four genes of aphids and used topical RNAi to interfere with these genes. This treatment affected the aphids’ growth and reproduction, caused mortality, and is harmless to their natural enemy, the ladybird beetles (*Propylea japonica*). The study showed that this gene-silencing method could be a strong tool for controlling pests. The contribution of this study is to assess the effectiveness of topical RNAi against a species of great agricultural interest, the cotton–melon aphid, while demonstrating harmlessness to the natural enemy, the ladybird beetles.

## 1. Introduction

The cotton–melon aphid (*Aphis gossypii* Glover), a member of the Aphididae family in the order Hemiptera, is a globally distributed piercing–sucking pest [1,2]. It attacks a wide range of plants, and causes severe damage to many important crops, including cotton, cucumber, pumpkin, watermelon, and melon [1,3]. The cotton–melon aphid feeds on plant phloem sap and secretes honeydew, which fosters mold growth [4]. Additionally, it is known to indirectly transmit over 100 plant viruses, significantly reducing crop yield and quality [5]. Chemical insecticides have long been widely used to manage cotton–melon aphids [6]. However, this approach has rapidly led to severe resistance development [4,7,8]. Controlling cotton–melon aphids remains a global challenge due to their complex life cycle, diverse reproductive strategies, variable coloration [9], and high resistance to multiple insecticides [3,4,7,10]. Therefore, there is an urgent need for a simple effective alternative technology, and RNA interference (RNAi) presents a promising solution.

RNAi is a post-transcriptional gene regulatory mechanism where double-stranded RNA (dsRNA) specifically silences target genes. This phenomenon was first discovered by Fire et al. in the nematode *Caenorhabditis elegans* [11]. In recent years, RNAi has become a powerful molecular tool for silencing essential gene transcripts in various insect orders, including Coleoptera, Hemiptera, Diptera, Hymenoptera, and Lepidoptera [12,13,14,15,16]. Thanks to its high sequence specificity, RNAi has emerged as an innovative strategy for managing numerous pest species [17]. Furthermore, the first RNA-based biopesticide received registration approval from the U.S. Environmental Protection Agency (EPA) [18], marking a significant milestone for RNAi technology in plant protection. However, this advancement also introduces new technical challenges. One of the major challenges for effective dsRNA-mediated pest management is the development of simple, cost-effective, and efficient dsRNA delivery systems [19,20].

Currently, microinjection, which demonstrates high RNAi efficiency, is widely employed; however, it is technically challenging and requires precise handling. In contrast, the feeding method enables pests to actively ingest sufficient dsRNA. Innovative approaches, such as host-induced gene silencing (HIGS) [21], virus-induced gene silencing (VIGS) [22], and nanocarrier-mediated delivery [19], ensure RNAi efficiency; yet, they fail to quantify the amount of dsRNA ingested [23,24]. A convenient and effective RNAi method based on topical dsRNA delivery is time-efficient [18,25,26,27]. This method has the potential to significantly influence RNAi-based gene function studies and the identification of candidate genes for RNAi-mediated pest control strategies. The efficacy of this delivery system has been demonstrated in the context of the Asian citrus psyllid *Diaphorina citri* and pea aphid *Acyrthosiphon pisum* [25,26]. However, the optimization and evaluation of this delivery method for the cotton–melon aphid has not been studied.

Based on previous studies, we set four genes, V-type proton ATPase subunit E (*ATPE*), Inhibitor of Apoptosis 1 (*IAP*), Cathepsin-L (*Cat*), and branched-chain amino acid aminotransferase (*ilvE*). These four genes were used as target genes to evaluate topical RNAi for the cotton–melon aphid. Two genes, *ATPE* and *IAP*, are essential for the energy supply and programmed cell death of insects and are also required for their survival [28]. These genes have been targeted for pest control [29,30]. The gene *Cat* is a key component of intestinal digestive enzymes in several invertebrate species. It is expressed in various insect body parts with diverse functions, making it a promising target for pest control [31]. The gene *ilvE*, encoding branched-chain amino acid aminotransferase, plays a key role in amino acid synthesis between aphids and the endosymbiotic bacterium *Buchnera aphidicola* [23,32]. Notably, *ilvE* is highly conserved across aphid species, highlighting its potential as a target for anti-aphid RNAi interventions. However, it is not clear whether these genes can be used as target genes for the control of cotton–melon aphid.

Furthermore, topical RNAi is employed as an exogenous dsRNA method, and whether it has adverse ecological effects on non-target organisms is one of the important questions that must be addressed prior to its commercialization [29,33,34]. While a substantial body of research has yielded fundamental insights into the non-target effects of RNAi, there is a paucity of studies that have directly examined the impact of RNAi on non-target organisms, particularly beneficial insects [33]. The ladybird beetle, *Propylea japonica* (Coleoptera: Coccinellidae), is a major predator of aphids, whiteflies, and other pests and is widely distributed in Asia [35]. It is a voracious predator that rapidly adapts to elevated temperatures and insecticide exposure, rendering it an ideal biological control agent with strong commercial potential [36]. In this study, *P. japonica* was employed as a model to investigate the non-target effects of RNAi for aphid control.

In this study, we performed RNAi in cotton–melon aphids using topical dsRNA delivery and assessed its efficiency in silencing four target genes with distinct functions: *ATPE*, *IAP*, *Cat*, and *ilvE*. We also evaluated the relevant biological performance of aphids following RNAi treatment. In addition, we offered topical RNAi-treated aphids as food to the predatory ladybird beetle to assess non-target risk.

## 2. Materials and Methods

### 2.1. Aphid Rearing

Cotton–melon aphids were long-term indoor reared populations of aphids (reared for more than 30 generations) under laboratory conditions (25 ± 1 °C; 75% RH; 16:8 L:D) using insecticide-free zucchini seedlings.

### 2.2. Total RNA Extraction and cDNA Synthesis

Total RNA was extracted from aphids using RNAiso reagent (TaKaRa, Maebashi, Japan) according to the manufacturer’s instructions, and the RNA was analyzed using electrophoresis on a 1.5% agarose gel. A Nano-Drop 2000 spectrophotometer (Thermo Scientific, Waltham, MA, USA) was used to test RNA quality and concentration. Complementary DNA (cDNA) synthesis was performed from 1 μg total RNA according to the instructions of the HiFiScript All-in-one RT Master Mix Kit (CWBIO, Taizhou, China). The resulting cDNA was stored at −20 °C for subsequent experiments.

### 2.3. Targeted Gene Cloning and Quantitative Real-Time PCR (RT-qPCR)

NCBI tool Primer-Blast (https://www.ncbi.nlm.nih.gov/tools/primer-blast/, accessed on 1 May 2024) was used to design specific primers (Table S1) according to our *A. gossypii* transcriptome data (Accession Number: SRP552655). The target gene fragment of *A. gossypii* was amplified by PCR from *A. gossypii* cDNA using the specific primers mentioned above. The resulting DNA fragment was ligated into the pMD19-T vector. The approximate length of the insert fragment was then determined by colony PCR, and the PCR products were sequenced. The cDNA sequences of the four genes were then uploaded to GenBank (*IAP*: PQ845428, *ATPE*: PQ845429, *Cat*: PQ845430, *ilvE*: PQ845431). To quantify the transcript levels of the target genes at different developmental stages of the aphid, RT-qPCR was performed. The qPCR specific primers (Table S1) were designed as described above, and standard curves were constructed using gradient dilutions of the cDNAs to check amplification efficiencies and cycling thresholds. Gene transcription was quantified by the 2^−ΔΔCt^ method using *EF1α* as reference gene [35].

### 2.4. Preparation of dsRNA

Using cDNA obtained from adult aphids, dsRNA regions were predicted using siDirect 2.1 (http://sidirect2.rnai.jp/, accessed on 20 June 2024), specific amplification primers were designed using the NCBI tool Primer Blast, and the T7 RNA polymerase promoter was added to the forward and reverse primer as required for dsRNA synthesis (Table S1). The primers with the T7 promoter were used to obtain dsRNA synthesis templates by PCR from cDNA, and dsRNA was subsequently synthesized using the T7 RiboMAX^TM^ Express RNAi System Kit (Promega, Madison, WI, USA) according to the manufacturer’s protocol. The concentration and quality of each dsRNA sample were verified using the Nano-Drop 2000 spectrophotometer and electrophoresis (Thermo Fisher, Waltham, MA, USA) on a 1.5% agarose gel.

### 2.5. RNAi and Aphid Performance

Based on pea aphid topical RNAi [26], the following optimizations were made: Adult aphids were collected from zucchini leaves, and 0.5 μL of dsRNA solution was applied to their dorsal surface using a 0.1–2 μL micropipette. The dsRNA delivery was considered complete after the surface solution had fully dried (approximately 15 min) [27]. Treated aphids were then transferred onto freshly excised zucchini leaves, with the petioles wrapped in moist cotton to maintain leaf turgidity and placed on a 1% agar medium in a Petri dish (100 mm × 15 mm). According to a previous review [24], the dsRNA solution concentrations were set at 100, 200, 500, 1000, and 2000 ng/μL, and the control was the same concentration of dsGFP solution. The number of dead aphids, the number of newborn aphids and their body weights were counted after 72 h of treatment. To assess the efficiency of the RNAi, surviving aphids were collected for qPCR at 12 h, 36 h, and 72 h. Forty aphids were used as a biological replicate, and at least three biological replicates were set for each treatment.

### 2.6. Risk Assessment of the Non-Target Effect of Insect Predator P. japonica

Topical RNAi-treated cotton–melon aphids were fed to hatchling first-instar larvae of ladybird beetles, while untreated cotton–melon aphids were fed as controls. Aphids treated with topical delivered dsRNA were used for feeding immediately after completion of dsRNA delivery. An equal number of aphids were provided to the larvae during each instar. The quantity offered increased progressively as the larvae grew, ensuring a consistently sufficient supply of aphids. The developmental duration and pupation rate of larvae, the pupation duration and emergence rate of pupae were recorded, newly emerged adults were collected, separated into males and females, and their body weights at the time of adults were recorded. Fifty ladybird beetle larvae were set up in each treatment, and each larva was individually reared in a glass tube (20 mm diameter × 50 mm height), the mouth of which was sealed with a sponge ball to ensure air permeability and to prevent ladybird beetles from escaping.

### 2.7. Statistical Analysis

Data were analyzed using SPASS 22.0 (IBM, Armonk, NY, USA) presented as the mean ± standard error, using the Kolmogorov-Smirnov test and Levene’s test (residuals normal distribution and chi-square of error variance) to ensure that the assumptions of parametric analyses were met. Age-specific expression profiles of target genes were analyzed using one-way ANOVA and Tukey post-hoc tests. For the RNAi efficiency test, the relative expression of the genes was first normalized against the expression of the reference gene, and the RNAi efficiency was calculated using the following formula: RNAi efficiency = [(Relative expression of the target gene in the ds*GFP* control—Relative expression of the target gene in the target-gene-specific dsRNA treatment)/Relative expression of the target gene in the ds*GFP* control] × 100%. Data on gene expression levels, mortality, fecundity, and body weight of aphids after RNAi were statistically analyzed using Student’s *t*-test. The Mann–Whitney *U* test was used to analyze ladybird beetle larval developmental and pupation duration, and the chi-square test was used to analyze larval pupation rates and pupal emergence rates. The one-way ANOVA with LSD test was used to analyze the body weight of female and male. For all experiments, *p* < 0.05 was considered significant.

## 3. Results

### 3.1. Instar-Specific Expression Profiles of Target Genes

**Figure 1:**
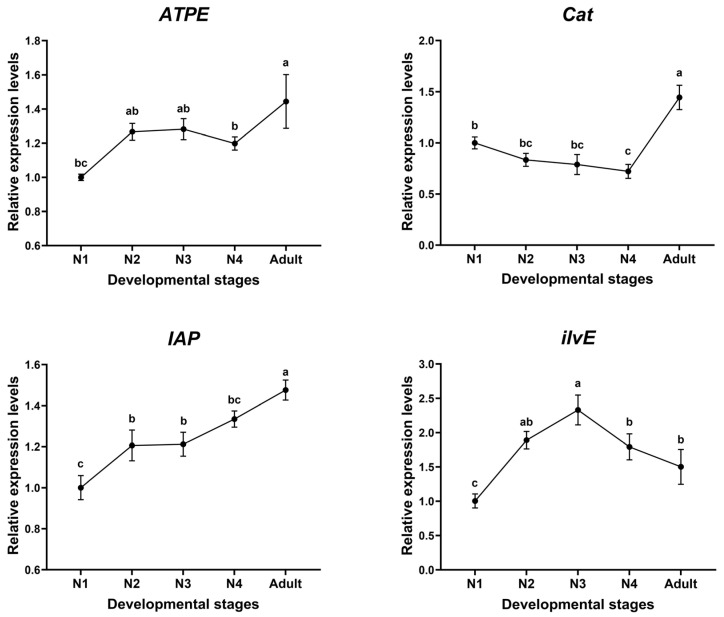
Relative expression pattern of four target genes at different developmental stages of *A. gossypii*. Different letters above each bar indicate significant difference determined by ANOVA with Tukey HSD test. Data are means ± standard error. The significance level was indicated as *p* < 0.05. N1: first instar; N2: second instar; N3: third instar; N4: fourth instar.

The relative mRNA abundance of *ilvE*, *ATPE*, *Cat,* and *IAP* genes in each developmental stage of aphids was examined using RT-qPCR (Figure 1). The four target genes were expressed in all developmental stages of aphids, and the expression profile of each gene was different. The highest expression of *ATPE* (*F*_4,10_ = 11.86, *p* < 0.001), *Cat* (*F*_4,10_ = 35.72, *p* < 0.001), and *IAP* (*F*_4,10_ = 26.41, *p* < 0.001) was found in the adult stage. There was no significant difference in the expression of *ATPE* among nymphal instars, whereas the expression of *Cat* declined with the development of nymphal stages, and that of *IAP* showed an increasing trend with the development of aphids. The expression of *ilvE* was significantly higher in the third instar than in the other instars (*F*_4,10_ = 20.82, *p* < 0.001), and there was no significant difference in the expression of the other developmental stages.

### 3.2. RNAi Efficiencies of Topical RNAi in Target Genes

**Figure 2:**
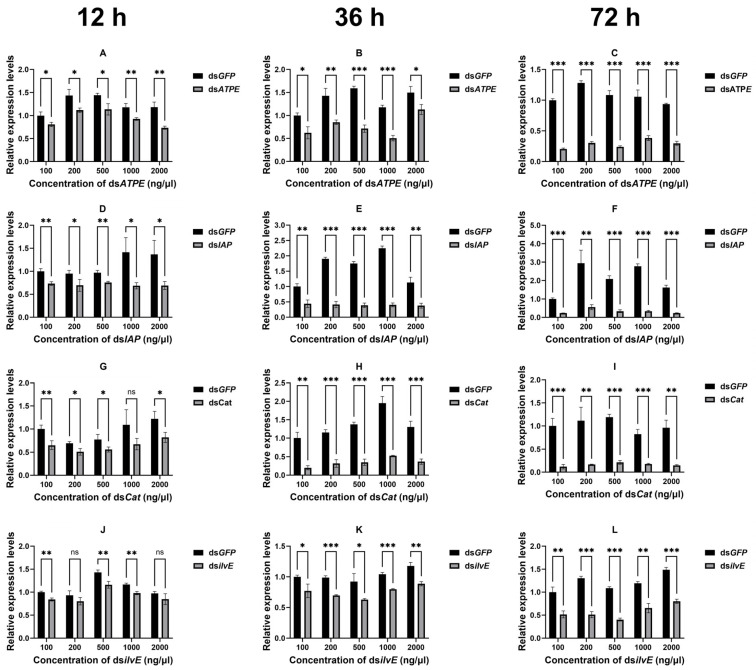
Silencing efficiency of four target genes of the aphid after 12 h (**A**,**D**,**G**,**J**), 36 h (**B**,**E**,**H**,**K**), and 72 h (**C**,**F**,**I**,**L**) of topical delivery of dsRNA at different concentrations. Data are means ± standard error. Data were analyzed by Student’s *t*-test. * *p* < 0.05, ** *p* < 0.01, *** *p* < 0.001, *ns* indicates no significant difference.

The silencing efficiency of aphid target genes after topical delivery of dsRNA was determined by RT-qPCR. The expression levels of relevant target genes were examined in adult aphids after 12, 36, and 72 h of topical delivery of dsRNA at different concentrations (100, 200, 500, 1000, and 2000 ng/μL) (Figure 2). The results showed that the expression of the vast majority of all genes was significantly decreased (all *p* < 0.05) at different times after the interference at different concentrations (Figure 2), except for ds*Cat* at 1000 ng/μL (12 h) (*df* = 4, *t* = 2. 057, *p* = 0.109) (Figure 2G) and ds*ilvE* at 200 ng/μL (12 h) (*df* = 4, *t* = 1.709, *p* = 0.163) and 2000 ng/μL (12 h) (*df* = 4, *t* = 1.732, *p* = 0.158) (Figure 2J). Our results showed that the silencing efficiency of RNAi was also increasing within 72 h after topical dsRNA delivery (Table S2).

### 3.3. Insecticidal Effect of Topical-Delivered RNAi on Aphids

**Figure 3:**
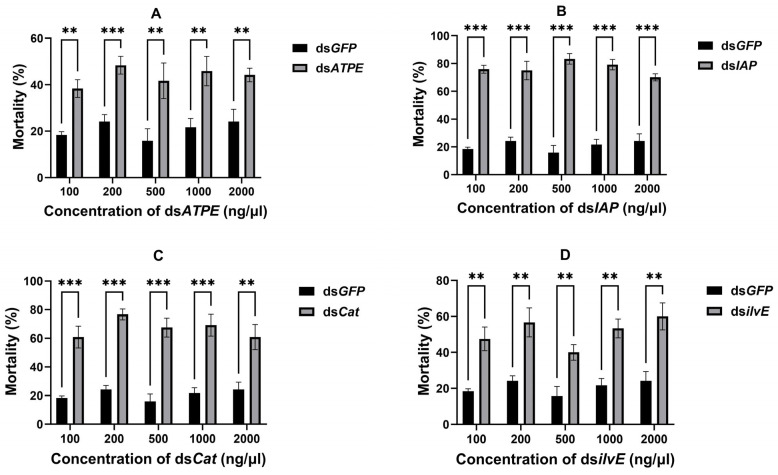
Aphid mortality after topical delivery of different concentrations of dsRNA for 72 h. (**A**) ds*ATPE*, (**B**) ds*IAP*, (**C**) ds*Cat,* and (**D**) ds*ilvE*. Data are means ± standard error. Data were analyzed by Student’s *t*-test. ** *p* < 0.01, *** *p* < 0.001.

Aphid mortality was counted 72 h after dsRNA delivery, and different concentrations of all four dsRNAs delivered via topical delivery resulted in significant aphid mortality (all *p* < 0.05) compared to the ds*GFP* control (Figure 3). The concentrations of each dsRNA with the highest mortality were (i) ds*ATPE*: 200 ng/μL (48.33%) (*df* = 4, *t* = 8.744, *p* < 0.001); (ii) ds*IAP*: 500 ng/μL (83.33%) (*df* = 4, *t* = 18.11, *p* < 0.001); (iii) ds*Cat*: 200 ng/μL (76.67%) (*df* = 4, *t* = 19.00, *p* < 0.001); and (iv) ds*ilvE*: 2000 ng/μL (60.00%) (*df* = 4, *t* = 6.799, *p* = 0.001).

### 3.4. Effects of Topical Delivered RNAi on Aphid Development and Reproduction

**Figure 4:**
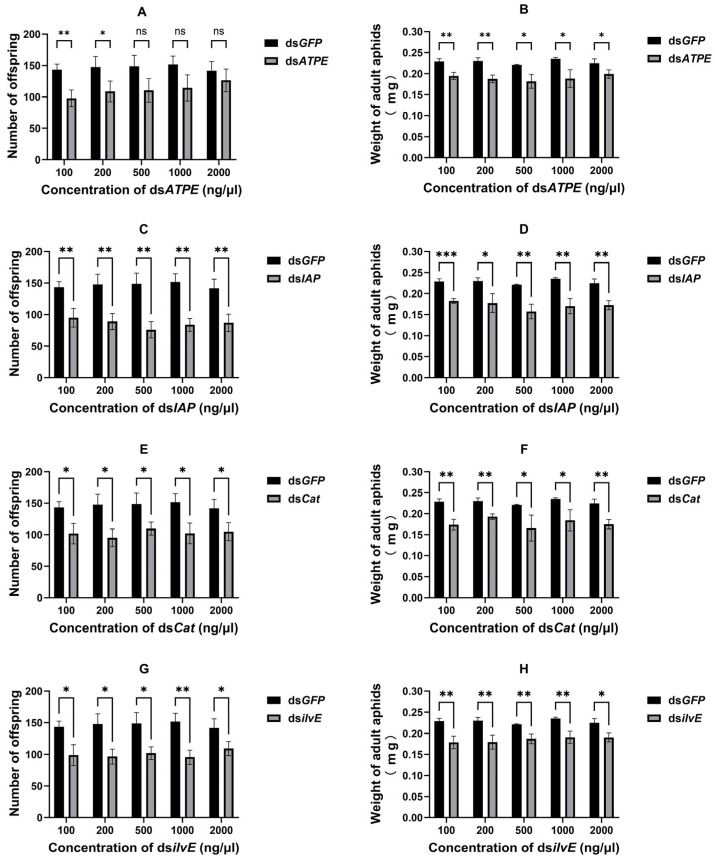
Effect of topical delivery of dsRNA on aphid fecundity [(**A**) ds*ATPE*, (**C**) ds*IAP*, (**E**) ds*Cat,* and (**G**) ds*ilvE*] and body weight [(**B**) ds*ATPE*, (**D**) ds*IAP*, (**F**) ds*Cat,* and (**H**) ds*ilvE*]. Data are means ± standard error. Data were analyzed by Student’s *t*-test. * *p* < 0.05, ** *p* < 0.01, *** *p* < 0.001, *ns* indicates no significant difference.

Most of the dsRNAs via topical delivery significantly reduced the fecundity of surviving aphids (all *p* < 0.05) (Figure 4A,C,E,G), except for ds*ATPE* at 500 ng/μL (*df* = 4, *t* = 2.605, *p* = 0.060), 1000 ng/μL (*df* = 4, *t* = 2.615, *p* = 0.059) and 2000 ng/μL (*df* = 4, *t* = 1.150, *p* = 0.314). In addition, the results showed that topical delivery of dsRNA significantly reduced the aphid body weight (all *p* < 0.05) (Figure 4B,D,F,H).

### 3.5. Effects of Topical RNAi-Treated of Aphids on the Predator Propylea japonica

Compared with the control group fed untreated aphids (larval duration: 6.08 days; pupal duration: 2.37 days), there were no significant differences in the larval developmental duration (ds*ATPE*: *p* = 0.150; ds*Cat*: *p* = 0.214; ds*IAP*: *p* = 0.542; ds*ilvE*: *p* = 0.683) and pupal developmental duration (ds*ATPE*: *p* = 0.953; ds*IAP*: *p* = 0.999; ds*Cat*: *p* = 0.416; ds*ilvE*: *p* = 0.063) of larvae fed on aphids treated with the four dsRNAs (Table 1).

Similarly, there were no significant differences in pupation (ds*ATPE*: χ^2^ = 1.010, *p* = 0.315; ds*IAP*: χ^2^ = 0, *p* = 1.000; ds*Cat*: χ^2^ = 1.010, *p* = 0.315; ds*ilvE*: χ^2^ = 0, *p* = 1.000) and emergence rates (ds*ATPE*: χ^2^ = 0.990, *p* = 0.320; ds*IAP*: χ^2^ = 0.344, *p* = 0.558; ds*Cat*: χ^2^ = 0.990, *p* = 0.320; ds*ilvE*: χ^2^ = 0, *p* = 1.000) among the four dsRNA treatment groups compared to the control group (pupation rate: 100%; emergence rate: 98%) (Table 1).

There was no significant difference in the body weight of their female (*F*_4,93_ = 0.5159; ds*ATPE*: *p* = 0.694; ds*IAP*: *p* = 0.465; ds*Cat*: *p* = 0.579; ds*ilvE*: *p* = 0.929) and male (*F*_4,141_ = 0.4408; ds*ATPE*: *p* = 0.812; ds*IAP*: *p* = 0.996; ds*Cat*: *p* = 0.504; ds*ilvE*: *p* = 0.513) adults compared to the control group (female: 5.70 mg; male: 5.13 mg) (Table 1).

## 4. Discussion

In RNAi-based gene function studies and pest management strategies, developing efficient, simple, and easy-to-apply delivery methods is crucial for identifying potential pest control targets [30,37,38,39]. In this study, we examined the expression patterns of four potential RNAi target gene (*ATPE*, *IAP*, *Cat*, and *ilvE*) across different developmental stages of cotton–melon aphid. We performed topical dsRNA delivery and evaluated the roles of these genes in aphid growth and reproduction. Our results showed that silencing these genes significantly affected the aphid survival, body weight, and fecundity, offering strong evidence for the use of topical dsRNA delivery in pest control and gene function studies.

The topical dsRNA delivery method used in this study was optimized based on the approach successfully used on pea aphids [26]. As the test subjects of this study, cotton–melon aphids, are much smaller than pea aphids, we reduced the volume of the dsRNA solution applied to aphids to 0.5 μL. The rationale behind our topical delivery method is that aphids efficiently absorb dsRNA through the body wall in a short time. Subsequent results demonstrated that dsRNA effectively reduced the relative expression of the target genes. Exposing the largest part of the body surface area of the whole aphid to the dsRNA solution aligned better with field practices and may serve as a useful reference for future RNAi applications in pest control.

In this study, our optimized topical dsRNA delivery technique effectively mediated RNAi, leading to the silencing of aphid-associated genes. Testing of various dsRNA delivery doses revealed that approximately 50 ng of dsRNA per adult aphid (0.5 μL of 100 ng/μL dsRNA solution) was sufficient to induce gene silencing. Measurement of target gene expression at various time points after dsRNA delivery revealed a significant decrease as early as 12 h, with ds*ATPE* and ds*IAP* showing a consistent reduction in expression at all concentrations applied at 12 h. This effect may be related to the type of target genes and the design of the dsRNA fragments. At 36 h following dsRNA treatment, target gene expression was significantly reduced in all experimental groups. Our results showed that the efficiency of topical dsRNA delivery gradually increased over time, peaking at 72 h among the time points tested. The optimal interference efficiencies of the four dsRNAs under different conditions ranged from 63.0% to 88.1% [ds*ATPE*: 79.5% (100 ng/μL); ds*IAP*: 88.0% (1000 ng/μL); ds*Cat*: 88.1% (100 ng/μL); ds*ilvE*: 63.0% (500 ng/μL)]. In comparison, RNAi efficiencies for different genes in the pea aphid, as measured by the topical delivery method, ranged from 41.0% to 99.9% [26]. The genes tested in that study were different from those in this experiment, highlighting the importance of screening efficient RNAi targets. Previous studies have shown that topical RNAi could be successfully achieved in other aphid species (*Myzus persicae* and *Aphis citricidus*) [26]. Other important sap-sucking pests, such as the Asian citrus psyllid *Diaphorina Citri* have also achieved topical RNAi [25,40]. Interestingly, our results showed that increasing the concentration of applied dsRNA did not improve interference efficiency. This may be due to the limited ability of insects to efficiently uptake and process dsRNA into siRNAs. Although RNAi effectiveness is dose-dependent, this relationship may not be linear and may reach a saturation point beyond which increasing the dsRNA concentration does not enhance interference efficiency.

Different concentrations of dsRNA targeting all four genes significantly increased aphid mortality compared to the ds*GFP* control. The ds*IAP* application exhibited the highest insecticidal efficacy (83.33%). The *IAP* gene is a negative regulator of apoptosis, and its knockdown in insects induces a pronounced apoptotic phenotype, leading to insect death. Knockdown of the *IAP*1 gene in beetle *Tribolium castaneum* resulted in 91% mortality, compared to other delivery methods [41]. Application of ds*Cat* resulted in up to 76.67% mortality, consistent with the results of topical ds*Cat* delivery to pea aphids [42]. Despite the higher interference efficiency of ds*ATPE* at various concentrations and time points, its effect on aphid fecundity was not significant at higher concentrations, and its insecticidal effect (48.33% mortality) was insufficient for aphid control application. This is due to the fact that V-ATPase consists of 14 different subunits, which exist in different combinations, and it is possible that when the function of some subunits is lost, other isoform subunits can partially compensate for their function. Some studies have used ds*ATPD* + ds*ATPE* synergistically to obtain better insecticidal effects [30]. In addition, insect epithelial tissues almost always require V-ATPase to regulate various processes [43]. This suggests that topical delivery of ds*ATPE* may achieve higher silencing efficiency. The ds*ilvE* exhibited lower insecticidal efficiency (60.00%) compared with other three genes. This may be because *ilvE* is mainly expressed in aphid bacteriocytes, and the efficiency of topical dsRNA delivery to these cells may be lower. Previous studies used a feeding method to silence key genes involved in the metabolic relay of aphid symbiotic bacteria, leading to a significant decrease in aphid weight and reproduction [32,44]. Silencing of *ilvE* disrupts the metabolic relay, negatively affecting aphid growth and development [32]. Aphids and their symbiotic bacterium, *Buchnera*, have formed a long-term, stable, and mutually beneficial relationship [23]. The relay process in the nutrient synthesis pathway provides important evidence of co-evolution. We look forward to identifying other key genes related to the aphid symbiotic bacterium as potential targets for pest control in future studies.

In the non-target effect assessment experiment, aphids that had just finished dsRNA delivery were transferred to ladybird beetle feeding chambers. The target gene dsRNA remaining on the aphid’s body surface at this time will be ingested by the ladybird beetle into its body surface as it feeds on the aphid. This experimental design simulates the exposure of ladybird beetles to exogenous dsRNA, whereas in other studies, non-target effects have been assessed by other means such as direct feeding of dsRNAs formulated into ladybird beetle diets and by injection [29,33,34]. Based on our biological measurements of ladybird beetles, the dsRNA of the target gene of the cotton–melon aphid did not significantly affect ladybird beetles. This demonstrates the specificity of RNAi for the target species and its harmlessness to non-target species [27,29,33,34]. Based on the results of the current experiments, we plan to conduct field trials as our next step to directly observe the control effects on pest populations as well as on non-target species and to evaluate the stability and persistence of topical RNAi under different climatic, soil, and ecological conditions [45].

Although topical RNAi has shown great potential as a method of controlling pests, the suitability of this technology for different insect species is currently understudied. In order to better utilize this technology, it is necessary to delve deeper into the specific mechanisms involved in the uptake of dsRNAs by the body barrier, as well as the expression of genes that are involved in this process [46]. This will help us understand the molecular basis of topical RNAi responses and provide a theoretical basis for developing more effective pest management strategies. In addition, scientists are exploring multiple approaches to improve dsRNA stability and delivery efficiency. The application of chemical modifications (including ribose modifications and backbone modifications) and nano-delivery systems (including lipid nanoparticles and chitosan nanoparticles) have been shown to significantly enhance the environmental stability and cellular uptake of dsRNAs, which, in turn, enhances the efficacy of RNAi [47,48]. In addition, it has been shown that certain insect populations may be resistant to RNAi-based pest control measures [49]. For example, the StaufenC gene, a key factor in dsRNA processing, has been identified to be associated with RNAi pesticide resistance in Coleoptera [50]. However, there is a relative paucity of relevant research on other insect groups such as Hemiptera, which limits our overall understanding of resistance in this group and the development of coping strategies [51].

Despite significant progress in the field of RNA-mediated pest management in Hemiptera, several key challenges remain to be overcome. These include but are not limited to the lack of dsRNA persistence in the natural environment, the complexity of uptake and transport mechanisms in insects, and the limitations in the understanding of the systemic transmission pathways [48,52]. Nevertheless, RNAi technology remains an attractive tool for crop protection, considering its high specificity, low toxicity, and potential friendliness to non-target organisms [27,29,51,52]. Therefore, future research should focus on addressing the aforementioned challenges, while at the same time working to increase public awareness and support for the technology and ensure that it can successfully pass the regulatory review process [45,48]. Only in this way can the potential of RNAi technology in integrated pest management in agriculture be fully realized and the development of sustainable agriculture be promoted.

## 5. Conclusions

In conclusion, we investigated the use of common pest control targets—*ATPE*, *IAP*, and *Cat*—for topical dsRNA delivery in cotton–melon aphids, successfully achieving RNAi-mediated silencing of these genes. This silencing significantly impacted aphid’s body weight and reproduction and ultimately led to their death. The *ilvE* gene, involved in relay metabolism in the aphid’s symbiotic bacterium, was also silenced by topical RNAi, demonstrating insecticidal efficacy. Assessment of off-target effects on the predator *P. japonica* showed that topical RNAi against the cotton–melon aphid was harmless to the ladybird beetle. As key candidate genes are screened, RNAi targets involved in various biological processes will become more readily identifiable. Topical dsRNA delivery holds promise as a valuable tool for gene function studies, with potential for developing novel strategies to enhance RNAi-based aphid control.

## Figures and Tables

**Table 1:** Life table parameters of ladybird beetles *Propylea japonica* reared on cotton–melon aphids treated with RNAi.

Treatment	Larval Developmental Duration (d)	Pupation Rate (%)	Pupal Developmental Duration (d)	Emergence Rate (%)	Adult Fresh Weight (mg)	
					Male	Female
CK	6.08 ± 0.10 a	100 a	2.37 ± 0.05 a	98 a	5.13 ± 0.11 a	5.70 ± 0.10 a
ds*ATPE*	5.89 ± 0.09 a	98 a	2.35 ± 0.05 a	100 a	5.16 ± 0.10 a	5.65 ± 0.13 a
ds*IAP*	6.10 ± 0.08 a	100 a	2.37 ± 0.04 a	96 a	5.13 ± 0.09 a	5.81 ± 0.10 a
ds*Cat*	6.26 ± 0.07 a	98 a	2.30 ± 0.04 a	100 a	5.04 ± 0.10 a	5.62 ± 0.10 a
ds*ilvE*	6.18 ± 0.09 a	100 a	2.24 ± 0.04 a	98 a	5.21 ± 0.07 a	5.72 ± 0.06 a

Data are means ± standard error. Means in a column followed by the same lowercase letter are not significantly different (*p* > 0.05).

## Data Availability

The data that support the findings of this study are available from the corresponding author upon reasonable request.

## Supplementary Materials

The following supporting information can be downloaded at https://www.mdpi.com/article/10.3390/insects16030276/s1, Table S1: Primers used in this study; Table S2: RNAi efficiency at each time point after topical dsRNA delivery.
