# Supplementary material for: Topical RNA Interference Induces Mortality in the Cotton–Melon Aphid Aphis gossypii with No Adverse Effect on the Predator Propylea japonica
Source: Insects. 2025 Mar 5;16(3):276. doi: 10.3390/insects16030276 (PMC11943396; doi:10.3390/insects16030276)
Supplement: Supplementary file 1 [file insects-16-00276-s001.zip › insects-3431408-supplementary.pdf]

Table S1. Primer information

Gene Name	Purpose	Forward (5' to 3')	Reversed (5' to 3')	qRT-PCR primer amplification efficiency (%)
ATPE	PCR	ATTGGGCGTTGGTTGTTCCA	TCTGTTAGGATTGCGACCGA	
IAP	PCR	TCACTGGTTGGCCAGTATCA	GCAAAATTAACAGCCATACCACA	
Cat	PCR	CCGCCCAAAAGCATAAGCC	CCCATCCAGGTCCCCAACT	
ilvE	PCR	ATCGACAACGAGTGGCTACC	GCGGTCTTTCGTCCACTACT	
EF1α	qRT-PCR	GAAGCCTGGTATGGTTGTCGT	GGGTGGGTTGTTCTTTGTG	100.6%
ATPE	qRT-PCR	GATGCCGATGTTCAAAAACAAATCA	CCAGACGGCCCTTTTCAATG	106.2%
IAP	qRT-PCR	CTACCTCCTGCACAAATGGC	CCATTGGACGAAAAGGTGTTCC	104.5%
Cat	qRT-PCR	GGTGTAGCCATTGTTGGTTATGG	ACCACAAGATCCATCACCTCTG	97.3%
ilvE	qRT-PCR	AAATGACCCCGATAAGTGTGT	GGTGTGATGTATGGTTTCTGCC	99.1%
ATPE	RNAi	GGATCCTAATACGACTCACTATAGGTTTGAGCGGAAAGAGAAACAAG	GGATCCTAATACGACTCACTATAGGTGAGCAATCAGTTCCAATCGTG	
IAP	RNAi	GGATCCTAATACGACTCACTATAGGGGGTACATTCACTGGTTGGC	GGATCCTAATACGACTCACTATAGGACGTTTTCAATCGAGCACTGA	
Cat	RNAi	GGATCCTAATACGACTCACTATAGGCATAAGCCATCACCTGTGGGA	GGATCCTAATACGACTCACTATAGGACATGGGGTTTGTTAGATCCTT	
ilvE	RNAi	GGATCCTAATACGACTCACTATAGGGCACAGAAGGCACACTAGG	GGATCCTAATACGACTCACTATAGGGATACTGCTATGACTGCTGCT	

Table S2. RNAi efficiency at each time point after topical dsRNA delivery

Treatment	12h RNAi efficiency (%)	36h RNAi efficiency (%)	72h RNAi efficiency (%)
100 ng/μl ds*ATPE*	19.4	37.7	79.5
200 ng/μl ds*ATPE*	22.3	40.2	76.2
500 ng/μl ds*ATPE*	21.2	55.1	77.8
1000 ng/μl ds*ATPE*	21.4	56.9	63.7
2000 ng/μl ds*ATPE*	37.7	24.5	68.1
100 ng/μl ds*IAP*	26.8	56.0	76.9
200 ng/μl ds*IAP*	27.0	78.1	81.2
500 ng/μl ds*IAP*	22.1	77.7	84.0
1000 ng/μl ds*IAP*	51.6	82.0	88.0
2000 ng/μl ds*IAP*	49.5	62.4	85.6
100 ng/μl ds*Cat*	34.8	80.2	88.1
200 ng/μl ds*Cat*	26.2	72.4	85.0
500 ng/μl ds*Cat*	32.6	74.6	82.1
1000 ng/μl ds*Cat*	38.2	73.0	78.6
2000 ng/μl ds*Cat*	33.1	71.8	84.6
100 ng/μl ds*ilvE*	15.9	22.9	48.4
200 ng/μl ds*ilvE*	13.7	29.6	60.9
500 ng/μl ds*ilvE*	18.8	31.9	63.0
1000 ng/μl ds*ilvE*	16.0	23.6	45.2
2000 ng/μl ds*ilvE*	12.9	24.7	46.0
